# Rod and Cone Function Measured Objectively by Chromatic Pupil Campimetry Show a Different Preservation Between Distinct Genotypes in Retinitis Pigmentosa

**DOI:** 10.1167/iovs.64.11.18

**Published:** 2023-08-14

**Authors:** Carina Kelbsch, Melanie Kempf, Ronja Jung, Friederike Kortüm, Milda Reith, Laura Kuehlewein, Susanne Kohl, Torsten Strasser, Tobias Peters, Helmut Wilhelm, Barbara Wilhelm, Krunoslav Stingl, Katarina Stingl

**Affiliations:** 1 University Eye Hospital, Centre for Ophthalmology, University of Tuebingen, Tuebingen, Germany; 2 Pupil Research Group at the Centre for Ophthalmology, University of Tuebingen, Tuebingen, Germany; 3 Center for Rare Eye Diseases, University of Tuebingen, Tuebingen, Germany; 4 Institute for Ophthalmic Research, Centre for Ophthalmology, University of Tuebingen, Tuebingen, Germany

**Keywords:** cone function, rod function, chromatic pupil campimetry, retinitis pigmentosa

## Abstract

**Purpose:**

Verifying whether specific genotypes causing retinitis pigmentosa (RP) show differences in the preservation of rod and cone function measured by chromatic pupil campimetry (CPC).

**Methods:**

Sixty-three RP eyes (37 male, 14–58 years) were measured using CPC with specific photopic and scotopic protocols, and the relative maximal constriction amplitudes and latencies to constriction onset were analyzed per genotype (RP due to variants in *EYS*, *n* = 14; *PDE6A*, *n* = 10; *RPE65*, *n* = 15; *USH2A*, *n* = 10; and *RPGR*, *n* = 14). Correlation analyses between the pupillary responses were performed with age, full-field stimulus threshold (FST), and optical coherence tomography (OCT) for cones and rods, respectively, to the genotype.

**Results:**

Pupillary responses were most severely reduced in *RPE65*-RP. Patients with disease-associated variants in *EYS* and *USH2A* were accompanied with better-preserved rod function compared with the other subgroups, reaching statistical significance between *EYS* and *RPE65*. Cone function was statistically significantly correlated with age in *USH2A*-RP with an annual decline of 2.4%. Correlations of pupillary responses were found with FST but barely with the ellipsoid zone area in OCT. Latency was significantly more prolonged in *RPE65*-RP compared with the other genotypes for cones.

**Conclusions:**

Rod and cone function measured objectively by CPC showed a different preservation between genotypes in RP. However, heterogeneity inside the same genotype was present. CPC data correlated with FST, but structural OCT parameters seem to be limited indicators for photoreceptor function in RP. Prolonged time dynamics for cones in *RPE65* mutations suggest an impact on cone processing and might provide additional information in the evaluation of therapy effects.

Retinitis pigmentosa (RP) is one of the most significant inherited retinal dystrophies, characterized by a progressive degeneration of rod and subsequent cone photoreceptors and is a major cause of visual impairment and blindness in the Western world. RP is a heterogenous group of rod–cone dystrophies, and the course of the disease differs greatly interindividually and is—among other factors—dependent on the underlying genetic subtype. Currently, numerous mutations in more than 80 different genes are identified to cause the clinical phenotype of nonsyndromic RP.^1^ Defective or loss of proteins in phototransduction, the visual cycle, or ciliary connection influences the clinical manifestation in various ways. In this study, we examined a large cohort of around 60 patients with RP in total, comprising five specific genetic subgroups with mutations in *PDE6A* (affecting the phototransduction cascade; encoding for the rod photoreceptor catalytic α subunit of the cyclic guanosine monophosphate [cGMP] phosphodiesterase), *RPE65* (affecting the visual cycle; reisomerization of all-*trans*-retinyl esters to 11-*cis*-retinal in the retinal pigment epithelium), *RPGR* (affecting the connecting cilium; responsible for most X-linked RP), *USH2A* (ciliopathy; Usher syndrome or nonsyndromic RP), or *EYS* (believed to affect the interphotoreceptor matrix and the connecting cilium; encoding for a binding protein to the hyaluronic acid network; one of the largest retinal genes; responsible for most nonsyndromic autosomal recessive RP).^1^^–^^3^

For ophthalmologists taking care of patients with RP, morphologic retinal changes in RP are relatively precisely measurable, with spectral-domain optical coherence tomography showing reduction of the outer retinal layers. However, objective parameters to assess retinal function of rods and cones are more challenging to obtain. For instance, electroretinography has its limits, particularly in late stages of the disease, and reflects an electrical sum response of the whole retina. Visual acuity and perimetry are highly subjective and dependent on the patient’s compliance and level of attention, representing only daylight cone function. Rod visual field or rod dark-adapted measurements are psychophysical tests with a high value for rod sensitivity but again require patients’ cooperation and do not evaluate the number of rods available.^4^ Chromatic pupil campimetry (CPC) has the potential to bridge the gap in the objective local assessment of rods and cones, as it allows for their broadly separate evaluation. It has been shown that CPC, analyzing pupillary responses to monochromatic local light stimuli, can objectively and retinotopically estimate local rod and cone function in healthy individuals^5^ and also in patients with inherited retinal dystrophies, including RP.^6^^–^^9^ CPC, in particular, has demonstrated its ability to assess the efficacy of treatment options on rod and cone function.^6^ The objective of this study was to investigate the identification of independent local functional degeneration changes in rods and cones in the natural course of RP, in various genotype cohorts. We hypothesize that specific genotypes causing defects at different steps of the retinal visual cycle, all leading to the phenotype of RP, will result in varying levels of preserved rod and cone function as measured objectively by CPC.

## Methods

### Participants

Fifty-seven voluntary patients with genetically confirmed RP were included in the study after detailed information and written informed consent according to the tenets of the Declaration of Helsinki and approval by the local ethics committee. Depending on the underlying genotype, five subgroups were formed (*n* representing the number of eyes included): patients with biallelic variants in *EYS* (*n* = 14, mean age 42 ± 13 years), *PDE6A* (*n* = 10, mean age 41 ± 8 years), *RPE65* (*n* = 15, mean age 26 ± 9 years), *USH2A* (*n* = 10, mean age 31 ± 11 years), and hemizygous variants in *RPGR* (*n* = 14, mean age 33 ± 12 years). For detailed genotype information, please refer to Supplementary Table S1. Six patients with biallelic mutations in *RPE65* had both eyes measured during natural course follow-up and included with a time span up to over 15 months between the two eye measurements. Thus, in total, 63 eyes were included in the study (37 male, 26 female; age 14–58 years, mean 34 ± 12 years).

All participants underwent a thorough ophthalmologic examination in the outpatient clinic for inherited retinal diseases at the University Eye Hospital Tuebingen, including optical coherence tomography (OCT; Spectralis-OCT, Heidelberg Engineering GmbH, Heidelberg, Germany) and full-field stimulus threshold (FST; Diagnosys, Cambridge, UK; using blue and red stimuli with 0 dB set to 0.01 cd⋅s/m^2^) and chromatic pupil campimetry (CPC; custom-built pupillograph).

### Chromatic Pupil Campimetry

Pupillary responses as an outcome parameter for retinal function were elicited and recorded by CPC. In a completely dark and quiet room, specific monochromatic stimuli were presented on a 55-inch OLED monitor (LG organic light-emitting diode 55C7V; LG Electronics, Seoul, South Korea; Full HD 3840 × 2160 pixels) within the central 30° visual field at a fixed distance of 40 cm to the participant's eye (the stimulated eye was recorded, and the other eye was patched). An infrared video camera with a sampling frequency of 100 Hz recorded the pupillary responses precisely, and gaze-tracking was implemented with retinotopic correct stimulus presentation. For the objective evaluation of primarily cone function, an L-cone–favoring photopic protocol with red stimuli on a dim blue background was used after 5 minutes of light adaptation time to the dim blue background (41 test locations within the 30° visual field, baseline period 500 ms, stimulus radius 3°, stimulus duration 1 second, stimulus intensity 60 cd/m^2^, stimulus wavelength 620 ± 30 nm full width at half maximum, 1.7 × 10^−5^ watt, interstimulus interval 4.5 seconds). After a subsequent 20 minutes of dark adaptation, rod function was evaluated by a rod-favoring scotopic protocol with dim blue stimuli on a completely dark background (33 test locations, baseline period 500 ms, stimulus radius 5°; stimulus duration 100 ms; stimulus intensity 0.01 cd/m^2^; stimulus wavelength 460 ± 30nm full width at half maximum, 2.1 × 10^−8^ watt, interstimulus interval 2.5 seconds). Stimuli were automatically repeated in case of lost gaze-tracking or strong artifacts (e.g., blinking during stimulus presentation) or if at least 90% of the initial pupil diameter was not reached again.

The method and protocols have been previously described in more detail and were validated, including the presentation of a test–retest reliability profile in a healthy control cohort^5^^,^^7^ and a cohort with inherited retinal diseases.^6^^,^^8^^,^^10^

### Data Analysis and Statistical Evaluation

Artifact elimination was performed by linear interpolation with an in-house created MATLAB software (The MathWorks, Inc., Natick, MA, USA; for more details, see Stingl et al.^9^). All pupillary traces were additionally manually checked and the interpolation optimized, if applicable. Analyses were performed in MATLAB and JMP (Version 16; SAS Institute, Cary, NC, USA). Pupillary traces are shown in relative values normalized to the baseline pupil diameter in accordance with the current expert standards in pupillography.^11^ The primary parameter was the relative maximal constriction amplitude (relMCA, in percentages). For the statistical comparison of relMCA between the genotypes, the central stimulus was used to reduce possible RP stage-dependent influences. Colored maps visualizing the extent of relMCAs throughout the tested 30° visual field are shown for cones and rods for each specific genotype, respectively. From the topographical three-dimensional presentation of these maps, based on all relMCAs of all stimulus locations, the “volume” was calculated and used for linear regression analyses with age, FST, and OCT data. The volume is hence a functional pupillary sum parameter based on all relMCAs (cone/rod function volume, in % * (deg)^2^).

For time dynamics, the latency to constriction onset (in ms) was analyzed, calculated as the time from the beginning of stimulus presentation until the time of intersection of linear fitting curves through the descending part of the pupillogram and the linear fitting curves through the baseline, respectively. Unreliable data of a latency calculation <150 ms or >1000 ms were automatically excluded and also if the absolute pupil constriction amplitude did not reach at least 0.1 mm. In potentially error-doubtful cases of an absolute constriction amplitude of subtle 0.1 to 0.2 mm and latencies of <200 ms and >700 ms, the respective pupillograms were automatically visualized and manually validated for accurate latency determination. To further improve the precision of the latency calculation, particularly in cases of only small residual pupil responses at all, we calculated the latencies from the mean pupil responses per eccentricity.

OCT area was calculated from the horizontal ellipsoid zone (EZ; in µm):

$$\begin{array}{c} OCT\;area=3.14\;x\;{\left(\frac{horizontal\;EZ}{2}\right)}^{2} \end{array}$$

The horizontal EZ was manually determined from the OCT data by a retina specialist with longstanding experience in inherited retinal dystrophies (KS). In four patients with *RPE65*-RP, the EZ was not determinable with sufficient accuracy, and therefore the recordings were not included in the data analysis. FST data were only available for the cohorts with biallelic disease-associated variants in *EYS*, *RPE65*, and *USH2A*.

For statistical comparisons of relMCA and latency per genotype, ANOVA and linear effects models were performed, followed by post hoc *t*-tests. Data are reported in least squares (LS) means, with the α level set to 0.05.

## Results

**Figure 1.**
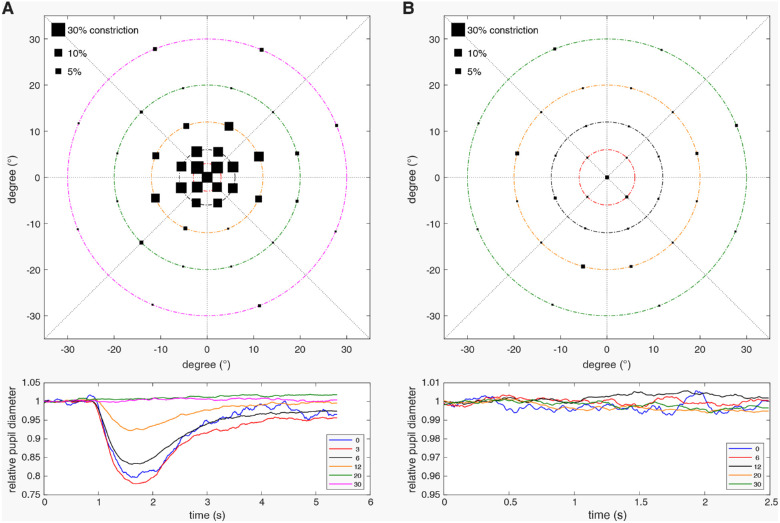
Pupillary responses measured by chromatic pupil campimetry for a patient with RP who has variants in *PDE6A* revealing preserved cone function within the central 12° of the visual field (**A**) and nonrecordable rod function with only discrete pupillary reaction noise (**B**). The sizes of the *black squares* per location in the *upper* graphs represent the amount of the relative maximal pupil constriction amplitudes referenced to the baseline pupil diameter. The *lower* graphs show the averaged normalized pupillary traces per eccentricity from 0° to 30° stimulation of the visual field.

The typical pupillary results measured by CPC for patients with RP (in this example due to variants in *PDE6A*) are shown in Figure 1 and display nonrecordable cone function in the periphery but preserved cone function in the central visual field and nonrecordable rod function in the entire tested field with only discrete pupillary reaction noise.

**Figure 2.**
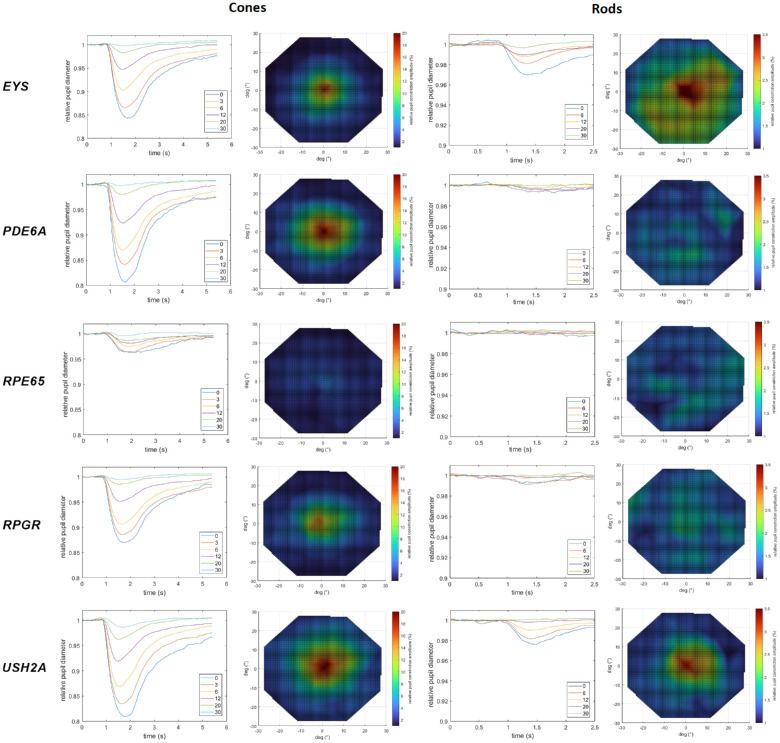
Averaged normalized pupillary traces per eccentricity from 0° central to 30° stimulation and color maps of the relative maximal constriction amplitudes in percentages (increasing pupillary responses from *blue* [low responses] over *green* and *yellow* to *red* [high responses] color) for the five different genotypes, respectively (*EYS*, *n* = 14; *PDE6A*, *n* = 10; *RPE65*, *n* = 15; *RPGR*, *n* = 14; and *USH2A*, *n* = 10). The *left column* shows the results for the cone-favoring protocol and the *right column* those for the rod-favoring protocol.

Figure 2 shows the averaged normalized pupillary traces selectively for cones and rods per eccentricity from central 0° to 30° peripheral stimulation of the visual field and corresponding color maps of the relMCAs for the five different genotypes, respectively.

**Figure 3.**
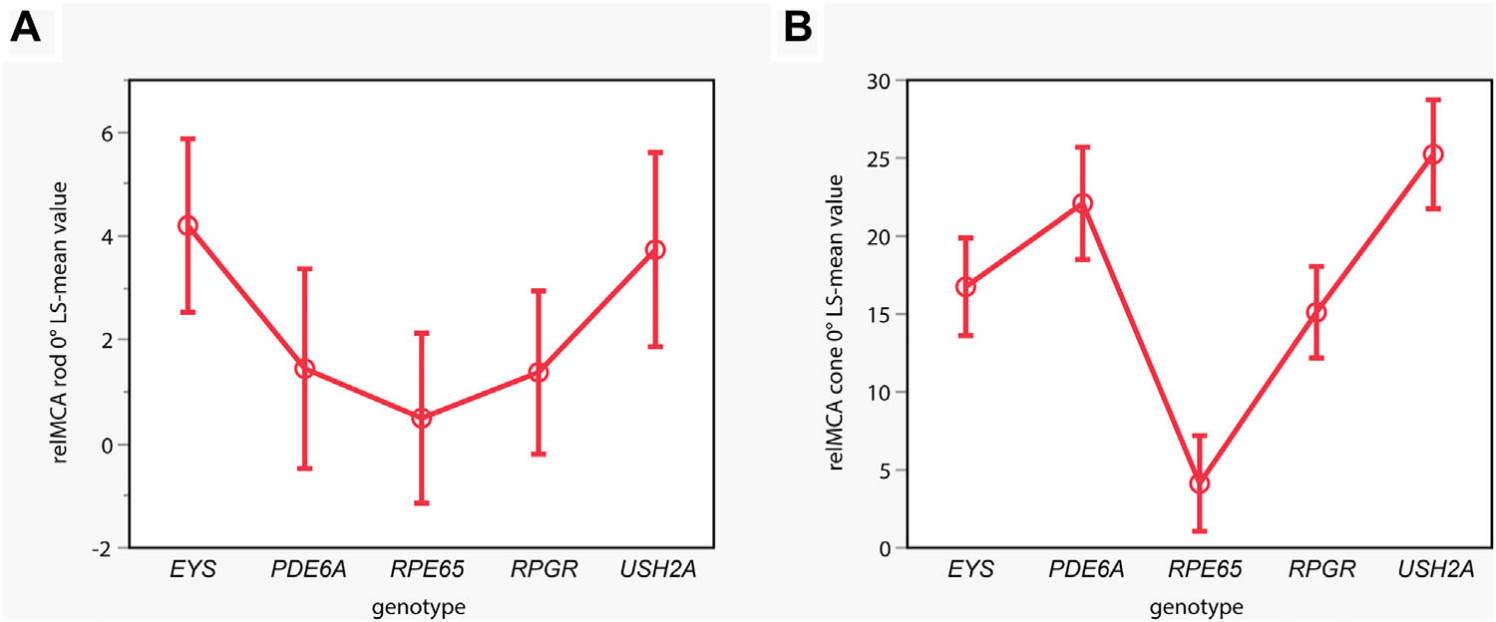
Statistical analysis comparing the LS mean values of the relMCA in percentages at central stimulation for the rod-favoring scotopic protocol (**A**; y-axis; stimulus radius 5°) per genotype (x-axis) of the linear effects model. Disease-associated variants in *EYS* and *USH2A* were accompanied by stronger pupillary responses with a statistically significant group difference between the *EYS* and *RPE65* groups. For the cone-favoring photopic protocol (**B**), statistically significant differences are shown with strongest central relMCA (stimulus radius 3°) in *USH2A*-linked RP, followed by the *PDE6A*, *EYS*, and *RPGR* groups and only residual cone function in the *RPE65* group. *EYS* (*n* = 14), *PDE6A* (*n* = 10), *RPE65* (*n* = 15), *RPGR* (*n* = 14), and *USH2A* (*n* = 10).

From the averaged data presented in Figure 2, in general, pupillary responses were most severely reduced in *RPE65*-RP. Patients with variants in *EYS* or *USH2A* had better-preserved rod responses than *RPGR*, *PDE6A*, and *RPE65* cases. Individual rod responses were evaluated to determine whether the group effect observed in *EYS* and *USH2A* cases was due to only a few patients with relatively well-preserved responses. Clearly detectable pupillary responses reflecting rod function were evident in 7 of 14 *EYS*, 6 of 10 *USH2A*, 6 of 14 *RPGR*, 3 of 10 *PDE6A*, and 1 of 15 *RPE65* cases. The effects model revealed a statistically significant effect of the genotype on central relMCA (stimulus radius 5°) for rods (see Fig. 3A), with a statistically significant group difference between the *EYS* and the *RPE65* group (*P* = 0.0325).

For cones, best-preserved central relMCA (stimulus radius 3°) was found in *USH2A*-linked RP (LS mean, SE; 25.24%, 1.75), followed by the *PDE6A* (22.09%, 1.80), *EYS* (16.75%, 1.56), and *RPGR* groups (15.11%, 1.47) and only residual cone function in the *RPE65* group (4.14%, 1.53). A likewise significant effect of the genotype on central relMCA was shown for cones: the *RPE65* group presented with statistically significantly lower cone function compared with all the other subgroups with *P* < 0.0001, respectively (see Fig. 3B). Furthermore, there was a statistically significantly better cone function in the *USH2A* group compared to the *RPGR* group (*P* = 0.0004) and the *EYS* group (*P* = 0.0066) and in the *PDE6A* group compared to the *RPGR* group (*P* = 0.0330).

Age had no confounding effect in the group analyses of central relMCA.

### Correlation of Pupillary Responses With Age in Dependence of Genotypes

**Figure 4.**
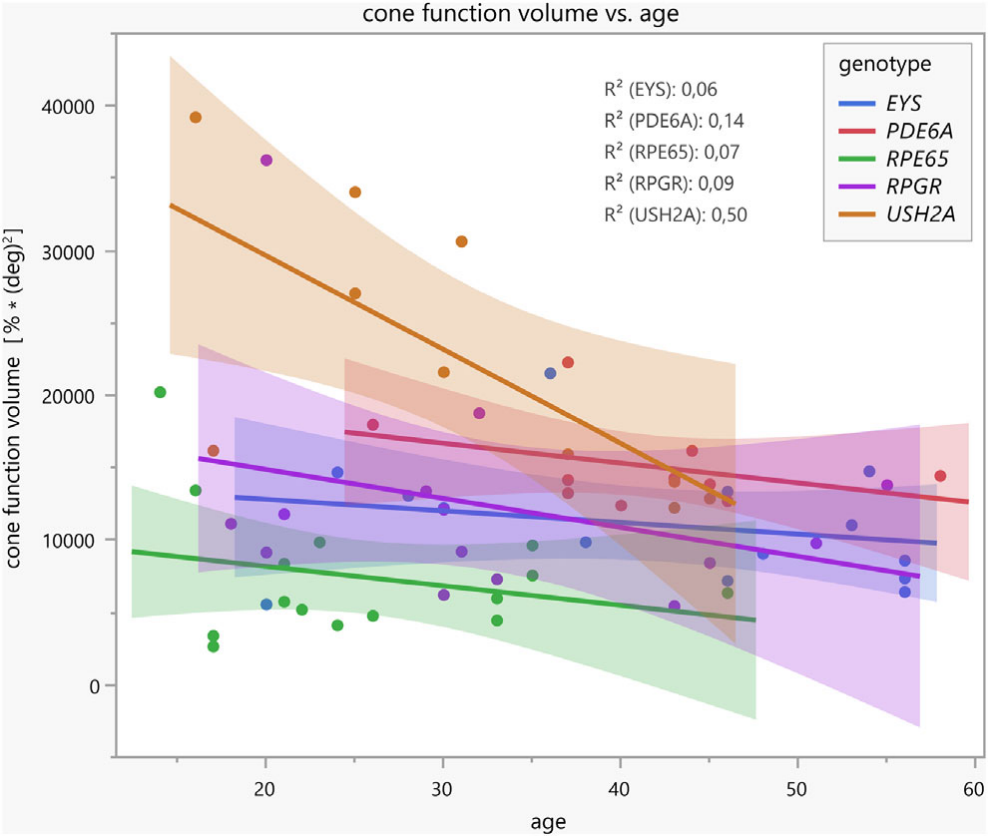
Linear regressions with confidence intervals between the cone function volume (functional pupillary sum parameter calculated of all normalized maximal constriction amplitudes to local stimuli throughout the 30° visual field measured by CPC with a cone-favoring protocol) and age, separately for the five different genotype groups. Patients with disease-causing variants in *USH2A* revealed the best-preserved cone function in younger ages with relatively steep, statistically significant decline over the years (*R*^2^ = 0.50; *F*(1, 8) = 7.97, *P* = 0.0224).

**Figure 5.**
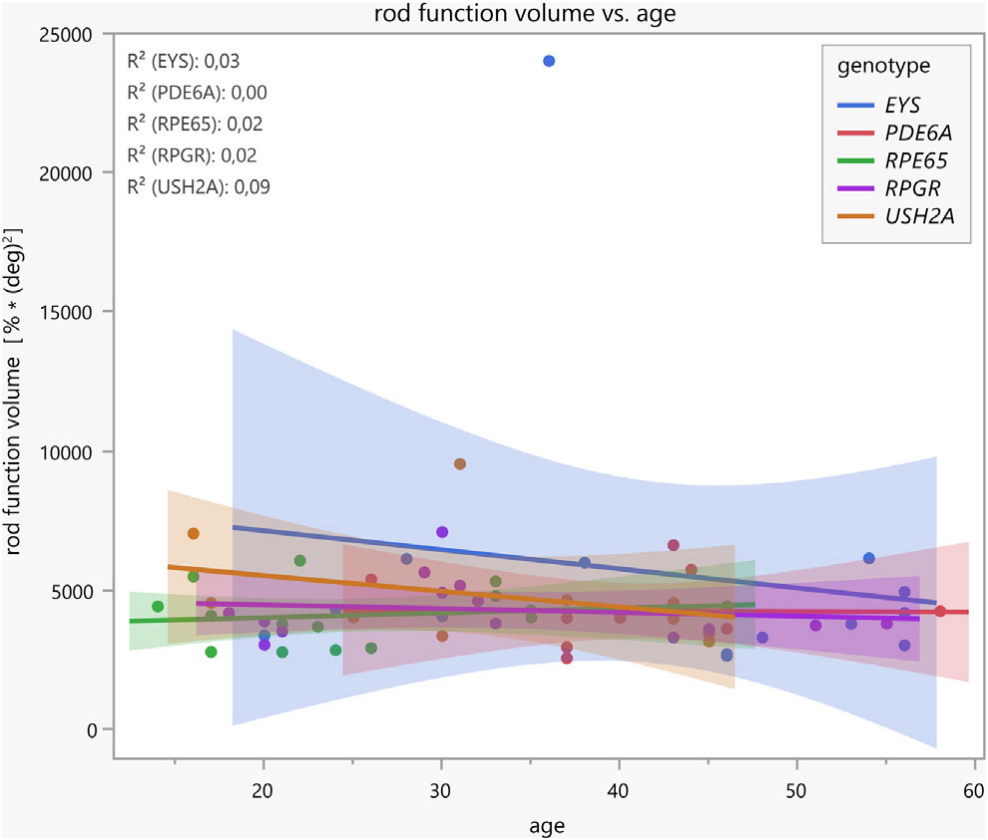
Linear regressions with confidence intervals between the rod function volume (functional pupillary sum parameter calculated of all normalized maximal constriction amplitudes to local stimuli throughout the 30° visual field measured by CPC with a rod-favoring protocol) and age, separately for five different genotype groups. Patients with variants in *EYS* and *USH2A* showed better-preserved rod function volumes in younger ages with subsequent nonsignificant decline with increasing age.

For correlations, the cone–rod function volume is used as a functional pupillary sum parameter based on all relMCAs. Linear regression analyses show a tendency of a decrease in cone function volume with increasing age (see Fig. 4). Patients with disease-causing variants in *USH2A* revealed the best-preserved cone function in younger ages, with a relatively steep, statistically significant decline over the years (*R*^2^ = 0.50; *F*(1, 8) = 7.97, *P* = 0.0224). The mean annual decline was −2.4% (confidence intervals, −4.4% to −0.4%), whereas the other four gene groups already showed lower cone function volumes in early ages with statistically nonsignificant further decline with increasing age. Figure 5 demonstrates the respective linear regressions of the residual rod function volume with increasing age, revealing no statistically significant correlations. Patients from the *EYS* and *USH2A* groups showed better-preserved rod function volumes in younger ages with subsequent nonsignificant decline with increasing age; individuals from the *PDE6A*, *RPE65*, and *RPGR* groups revealed only residual rod function volumes for all ages.

### Correlation of Pupillary Responses With FST

**Figure 6.**
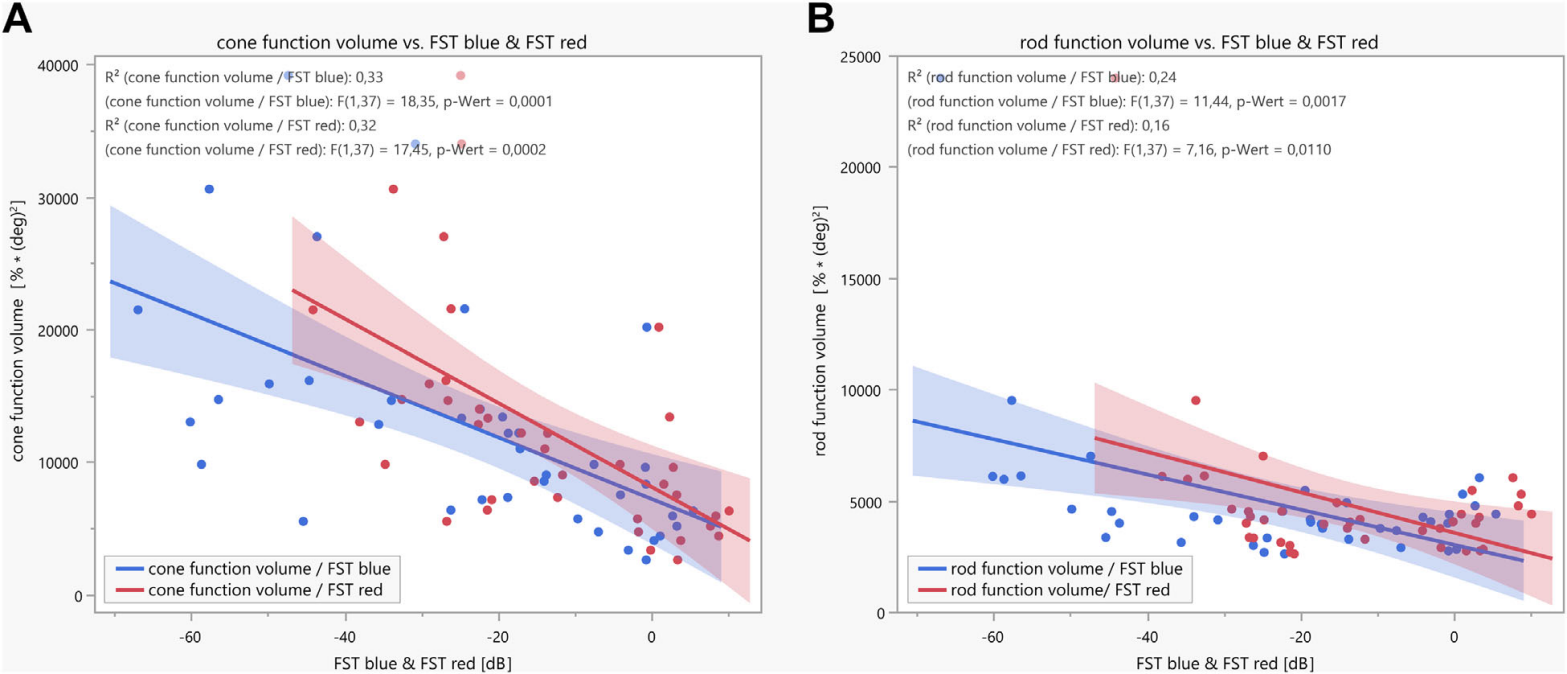
Linear regressions with confidence intervals between either cone (**A**) or rod (**B**) function volume, measured by CPC and calculated from all normalized maximal constriction amplitudes to local stimuli throughout the 30° visual field, with full-field stimulus threshold measurements (FST, *blue* and *red*, respectively). FST data were available for the *EYS*, *RPE65*, and *USH2A* cohorts (*n* = 39).

FST data using blue and red stimuli were available for the cohorts with disease-causing variants in *EYS*, *RPE65*, and *USH2A* (in dB; *n* = 39). There was a statistically significant weak correlation between both FST blue and FST red, with the cone function volume (Fig. 6A) and the rod function volume (Fig. 6B). For cone function volume, the parameters of the linear regression analyses were as follows: *R*^2^ = 0.33, *F*(1, 37) = 18.35, *P* = 0.0001 for FST blue and *R*^2^ = 0.32, *F*(1, 37) = 17.45, *P* = 0.0002 for FST red. For rod function volume, the parameters were *R*^2^ = 0.24, *F*(1, 37) = 11.44, *P* = 0.0017 for FST blue and *R*^2^ = 0.16, *F*(1, 37) = 7.16, *P* = 0.011 for FST red.

### Correlation of Pupillary Responses With OCT Area of the Ellipsoid Zone in Dependence of Genotypes

**Figure 7.**
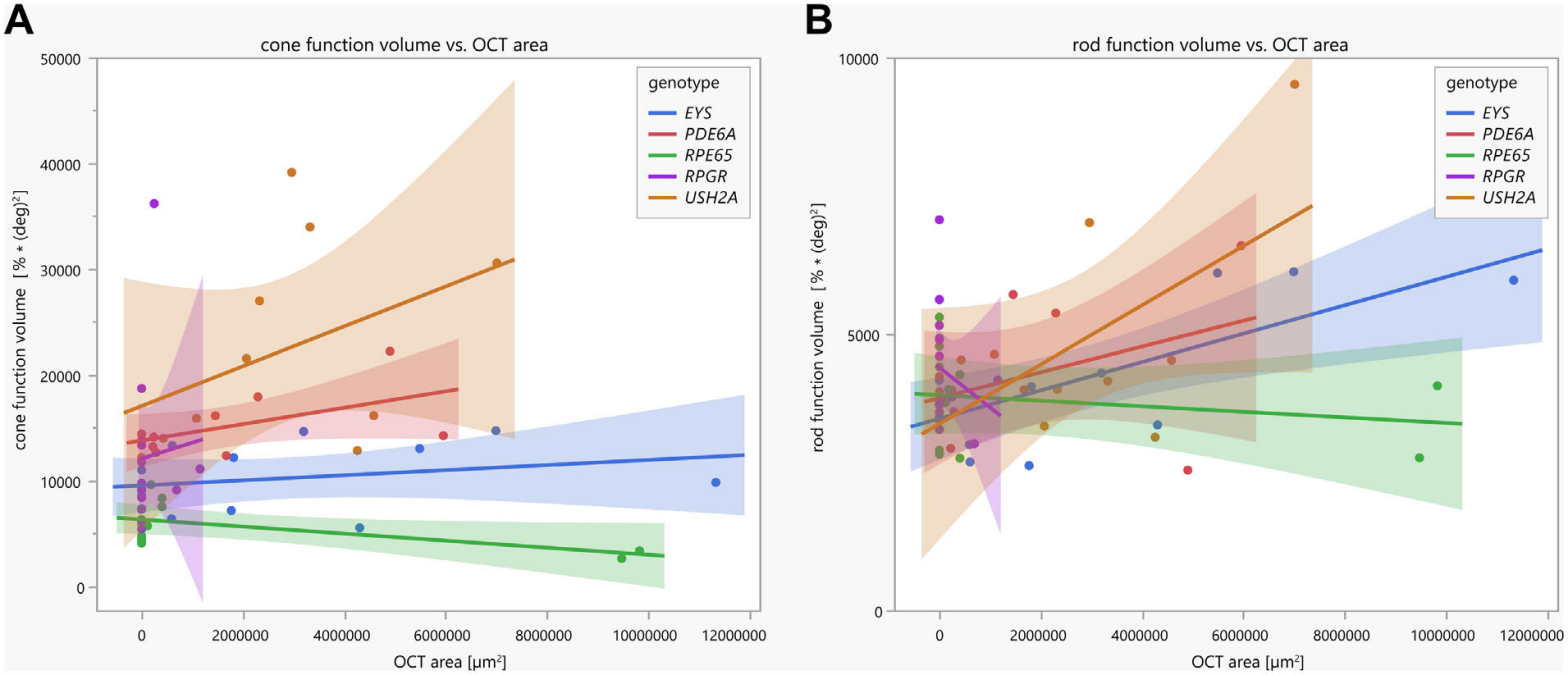
Linear regressions with confidence intervals between the cone/rod function volume (functional pupillary sum parameter calculated of all normalized maximal constriction amplitudes to local stimuli throughout the 30° visual field measured by CPC) and the OCT area (calculated from the preserved horizontal ellipsoid zone), separately for the five different genotypes and for cone function (**A**) and rod function (**B**), respectively.

Linear regression analyses between the area of preserved EZ in the OCT and the pupillary function volumes revealed statistical significance only for the rod function volume for the *EYS* group (*R*^2^ = 0.49, *F*(1, 11) = 10.78, *P* = 0.0073); see Figure 7.

### Time Dynamics

For rods, we provide no statistics, as we considered the latency calculation too error prone due to frequently only very subtle or nonrecordable pupillary rod responses in our cohorts, particularly in *RPE65-RP*. For cones, a significant effect on time dynamics was shown for the genotype and the eccentricity and a minor contribution of age. In general, increasing eccentricity was associated with correspondingly increasing latencies. Patients from the *RPE65* group showed significantly longer latencies for stimulation of the central 0°, 3°, 6°, and 12° compared with all the other genotype groups (see Table). At the eccentricity of 20°, the *RPE65* group still had significantly longer latencies than the *USH2A*, *EYS*, and *PDE6A* groups. Beyond that eccentricity, no more differences could be demonstrated. However, with increasing eccentricity, many of the participants showed only residual and/or spontaneous discrete noisy pupillary responses, which impeded good signals for a proper time dynamic analysis and reliable comparison, particularly at 20° and 30° eccentricities. As stated in the Methods section, all calculated latencies >1000 ms were excluded from the analysis as being obviously physiologically unlikely.

**Table.** Mean Latency to Constriction Onset for Cones per Eccentricity and Genotype Group

	*EYS*	*PDE6A*	*RPE65*	*RPGR*	*USH2A*	Significance
0°	305 ms	314 ms	439 ms	346 ms	308 ms	*RPE65* longer latency than all others;
						*P* = 0.0178 to 0.0002
3°	302 ms	319 ms	452 ms	353 ms	309 ms	*RPE65* longer latency than all others;
						*P* = 0.0006 to <0.0001
6°	313 ms	336 ms	495 ms	382 ms	322 ms	*RPE65* longer latency than all others;
						*P* = 0.0011 to <0.0001
12°	342 ms	365 ms	508 ms	433 ms	360 ms	*RPE65* longer latency than all others;
						*P* = 0.0437 to <0.0001
						*RPGR* longer latency than *EYS; P* = 0.0060
20°	414 ms	422 ms	591 ms	490 ms	419 ms	*RPE65* longer latency than *EYS, USH2A, PDE6A*
						*P* = 0.0180 to 0.0061
30°	499 ms	488 ms	603 ms	582 ms	458 ms	n.s.

Statistically significant differences are given with respective *P* values.

## Discussion

This study encompasses a large cohort of genetically confirmed patients with RP separately analyzed for five distinct genotypes with disease-causing variants in *EYS*, *PDE6A, RPE65*, *RPGR*, or *USH2A.* As the encoded defective or lost proteins play important roles in phototransduction, the visual cycle, or the photoreceptor ciliary connection, the clinical manifestation of RP is heterogeneous and, among other factors, dependent on the genetic subtype.^1^ The results of our cohort indicate a likewise different preservation of cone and rod function between the genetic subgroups measured objectively by CPC. Patients with disease-causing variants in *PDE6A* and *RPE65*, encoding components of the rod phototransduction cascade or the visual cycle, respectively, had the worst rod function in averaged (see Fig. 2) and individual data. However, ciliary dysfunction caused by variants in *EYS* and *USH2A* showed better-preserved rod function likewise in averaged and individual data. Cone function, instead, was best preserved in the *USH2A* and *PDE6A* groups. For the *USH2A* group, cone function was negatively correlated with age, and the other subgroups did not show this correlation as cone function was already heavily reduced in younger ages. It was previously described that patients with *USH2A*-RP annually decline in visual acuity around 2.6%, in visual field area around 7%, and for cone electroretinographic amplitude around 13.2%, with the acuity loss being slower and the other parameters being faster than for *RPGR* patients.^12^ The *USH2A* group in our data showed a reduction in cone function volume by approximately 2.4% within the visual acuity decline range reported by Sandberg et al.^12^ The missing age effect in the other subgroups is presumably due to the sample size, interindividual variability of disease impairment, and progressive degeneration. For instance, *RPE65*-related inherited retinal dystrophies are usually characterized by an early-onset retinal degeneration with progressive visual deterioration already in young ages. This seems to be relatively uniform, indicating that most missense mutations in these genes and proteins lead to loss of function.^13^ Analogously, pupillary responses measured by CPC were most severely reduced in *RPE65* cases compared with the other genotypes of our cohort, showing only very residual cone function and nearly extinguished rod function.

For testing photoreceptor psychophysics, FST is widely used in gene therapy studies as well as in recent postapproval real-life publications.^4^^,^^6^^,^^14^^,^^15^ Linear regression analyses between pupillary responses and FST revealed a significant but weak correlation between the two methods, hereby confirming the significance of both methods. The lower the threshold in FST, the better the measurable pupillary function volume response. CPC is proposed to measure retinal function based on the amount of residual responding photoreceptors in the stimulated area, whereas FST determines the threshold of perception, and thus CPC and FST display different complement retinal function.^7^

The remaining intact EZ area in OCT is one of the most used anatomic-based structural outcome parameters in clinical studies and seems to show low repeat variability.^16^ Therefore, we examined whether the morphology obtained by the OCT EZ area correlates with the retinal function measured by CPC, but no good correlation was found in our cohort: statistical significance of the linear regression analyses was only found for OCT area and rod function volume in the *EYS* group. We consider several factors for this discrepancy. The EZ represents structurally remaining photoreceptors, but the presence of cell bodies is not necessarily connected to adequate function. In *RPE65*-associated RP, such a pattern of structural–functional dissociation has been described by demonstrating relatively preserved photoreceptors in OCT, which were severely dysfunctional.^17^ Reversely, single cells with preserved function that are structurally not visible in OCT may be hypothesized to contribute. Correlations with other OCT parameters (e.g., outer retinal layer thickness obtained from a macular volume scan) were not performed as we considered it methodologically less robust or rather precise using predefined OCT areas for each patient with RP and not taking individually damaged structures into account. Moreover, OCT does not differentiate between rod and cone photoreceptors as does CPC with specifically designed scotopic and photopic stimulation protocols. Consequently, in our view, functional parameters are—in addition to structural parameters—of specific relevance in any therapeutic interventional study.

Furthermore, we addressed the question of altered time dynamics of the pupillary responses in RP by analyzing the latency to constriction onset, which can be considered a parameter for impaired processing of the retinal network from photoreceptors to ganglion cells in case of latency prolongation. In comparison to healthy individuals who revealed mean latencies of 277 ± 25 ms (range, 222–334 ms) with increasing latencies to more peripheral stimuli in photopic CPC in a former publication of our group,^5^ degenerated retina in RP was associated with longer latencies with a likewise increase from central to peripheral stimulation. Interestingly, in *RPE65*-RP, latencies for cones seem more prolonged than in the other genotype groups of our cohort. This suggests that *RPE65* might have more impact on cone function and processing and should be further examined (for an extensive updated review about *RPE65*, refer to Kiser^3^). This is of particular interest, as gene therapy with voretigene neparvovec is available for RP with biallelic disease-causing variants in *RPE65*.^14^^,^^15^^,^^17^ Consequently, individual changes after gene therapy with voretigene neparvovec might also occur in cone time dynamics. As a limitation, latency calculation is less robust and vulnerable to methodologic error in cases of only very subtle pupillary responses.

Our data indicate that with CPC, we obtain a reliable outcome parameter of local retinal function with the relMCA separated for rods and cones. In addition, with the latency, we obtain a parameter that measures the functional dynamics of the retina, which can offer significant insights in assessing the efficacy of any treatment.

Our results show differences of preserved cone and rod function dependent on the underlying genetic subtype of RP, but it is also obvious that retinal function varies considerably within one genotype group. One reason might be seen in the type of mutation (e.g., missense variants, nonsense variants, variants resulting in frame shift, splicing variants), leading either to some residual altered function or to *null* alleles with complete loss of the mutant protein or protein function. Additionally, genetic modifiers as well as exogenous factors can contribute to different phenotypes of RP, even in comparable genotypes. Further reasons might be seen in the natural variability of the extent of pupillary responses between participants.

### Limitations

Our focus was primarily to present clinically relevant information of novel examination procedures such as CPC and their correlation with routinely used clinical workup and read-outs. Although we included a considerable number of patients with RP, only a limited number (10–15) of eyes were analyzed per genetic subgroup. Therefore, there is no adequate age-matching that might interfere with group comparisons, and that has limited the scope of statistical testing and interpretations. Likewise, undoubtedly, in all genotypes, late-stage RP is associated with worse retinal function than earlier stages. However, age was relatively homogeneous between the groups, with the youngest mean age in the *RPE65* group revealing the worst pupillary responses and better-preserved pupillary responses in the *EYS* (rods) and *PDE6A* (cones) groups with on average older patients compared to the *RPGR* group, which is contradictory in case of a presumed only worse pupil response with increasing age.

A further limitation is the inclusion of both eyes in six participants with *RPE65*-RP as the two eyes of an individual are correlated. However, as these data were measured at different time points with spans up to 2 years between the measurements of both eyes and *RPE65*-RP being a fast-progressive disease, we felt that the benefit of including these data in such rare diseases outweighs this limitation.

## Supplementary Material

Supplement 1
