# Supplementary material for: Rod and Cone Function Measured Objectively by Chromatic Pupil Campimetry Show a Different Preservation Between Distinct Genotypes in Retinitis Pigmentosa
Source: Invest Ophthalmol Vis Sci. 2023 Aug 14;64(11):18. doi: 10.1167/iovs.64.11.18 (PMC10431211; doi:10.1167/iovs.64.11.18)
Supplement: Supplement 1 [file iovs-64-11-18_s001.pdf]

Mutation type	Subject ID	Variant 1	Variant 2	Segregation analysis available
EYS	EYS-01	EYS c.67dup;p.Thr23AsnfsTer3	EYS c.67dup;p.Thr23AsnfsTer4	
	EYS-02	EYS c.4350_4356del;p.Ile1451ProfsTer3	EYS c.4350_4356del;p.Ile1451ProfsTer3	
	EYS-03	EYS c.9405T>A;p.Tyr3135Ter	EYS c.9405T>A;p.Tyr3135Ter	
	EYS-04	EYS c.3024C>A;p.Cys1008Ter	EYS c.4350_4356del;p.Ile1451ProfsTer3	yes
	EYS-05	EYS c.2055T>A;p.Cys685Ter	EYS c.6714del;p.Ile2239SerfsTer17	yes
	EYS-06	EYS Deletion Exons 1-12	EYS Deletion Exons 3-4	yes
	EYS-07	EYS c.6714del;p.Ile2239SerfsTer17	EYS c.6714del;p.Ile2239SerfsTer17	
	EYS-08	EYS Deletion Exon 12	EYS Duplication/Inversion Exon 31	yes
	EYS-09	EYS c.4045C>T;p.Arg1349Ter	EYS c.8133_8137del;p.Phe2712Cysfs*3	
	EYS-10	EYS c.2194C>T;p.Gln732Ter	EYS c.2194C>T;p.Gln732Ter	
	EYS-11	EYS c.490C>T;p.Arg164Ter	EYS c.490C>T;p.Arg164Ter	
	EYS-12	EYS Duplication Exons 23+24	EYS Deletion Exon 15	yes
	EYS-13	EYS c.4045C>T;p.Arg1349Ter	EYS c.4350_4356del;p.Ile1451ProfsTer3	yes
	EYS-14	EYS c.8648_8649del;p.Thr2883MetfsTer6	EYS c.7723+1G>A;p.?	
PDE6A	PDE6A-01	PDE6A c.304C>A;p.Arg102Ser	PDE6A c.2053G>A;p.Val685Met	
	PDE6A-02	PDE6A c.1957C>T;p.Arg653Ter	PDE6A c.1956_1957ins20bp;p.Arg653Ter	
	PDE6A-03	PDE6A c.1705C>A;p.Gln569Lys	PDE6A c.1705C>A;p.Gln569Lys	
	PDE6A-04	PDE6A c.304C>A;p.Arg102Ser	PDE6A c.304C>A;p.Arg102Ser	
	PDE6A-05	PDE6A c.1065+2T>A;p.?	PDE6A c.1705C>A;p.Gln569Lys	yes
	PDE6A-06	PDE6A c.1957C>T;p.Arg653Ter	PDE6A c.1957C>T;p.Arg653Ter	
	PDE6A-07	PDE6A c.1957C>T;p.Arg653Ter	PDE6A c.1957C>T;p.Arg653Ter	
	PDE6A-08	PDE6A c.1957C>T;p.Arg653Ter	PDE6A c.2332_2335del;p.Asp778LeufsTer42	
	PDE6A-09	PDE6A c.998+1G>A;p.?	PDE6A c.998+1G>A;p.?	
	PDE6A-10	PDE6A c.304C>A;p.Arg102Ser	PDE6A c.1689C>A;p.His563Gln	yes

Mutation type	Subject ID	Variant 1	Variant 2	Segregation analysis available
RPE65	Lux-01	RPE65 c.1451G>T;p.Gly484Val	RPE65 c.1451G>T;p.Gly484Val	
	Lux-01	RPE65 c.1451G>T;p.Gly484Val	RPE65 c.1451G>T;p.Gly484Val	
	Lux-02	RPE65 c.1451G>T;p.Gly484Val	RPE65 c.1451G>T;p.Gly484Val	
	Lux-02	RPE65 c.1451G>T;p.Gly484Val	RPE65 c.1451G>T;p.Gly484Val	
	Lux-03	RPE65 c.1102T>C;p.Tyr368His	RPE65 c.1102T>C;p.Tyr368His	yes
	Lux-03	RPE65 c.1102T>C;p.Tyr368His	RPE65 c.1102T>C;p.Tyr368His	yes
	Lux-04	RPE65 c.1102T>C;p.Tyr368His	RPE65 c.1102T>C;p.Tyr368His	yes
	Lux-04	RPE65 c.1102T>C;p.Tyr368His	RPE65 c.1102T>C;p.Tyr368His	yes
	Lux-08	RPE65 c.208T>G;p.Phe70Val	RPE65 c.246-11A>G;p.?	
	Lux-10	RPE65 c.1451G>T;p.Gly484Val	RPE65 c.1451G>T;p.Gly484Val	
	Lux-10	RPE65 c.1451G>T;p.Gly484Val	RPE65 c.1451G>T;p.Gly484Val	
	Lux-11	RPE65 c.1451G>T;p.Gly484Val	RPE65 c.1451G>T;p.Gly484Val	
	Lux-12	RPE65 c.304G>T;p.Glu102Ter	RPE65 c.304G>T;p.Glu102Ter	yes
	Lux-12	RPE65 c.304G>T;p.Glu102Ter	RPE65 c.304G>T;p.Glu102Ter	yes
	Lux-13	RPE65 c.208T>G;p.Phe70Val	RPE65 c.246-11A>G;p.?	yes
USH2A	USH2A-01	USH2A c.653T>A;p.Val218Glu	USH2A c.11105G>A;p.Trp3702Ter	
	USH2A-03	USH2A c.13010C>T;p.Thr4337Met	USH2A Deletion Exon 23	yes
	USH2A-04	USH2A c.949C>A;p.Arg317= (Splice Defect)	USH2A c.15496A>G;p.I5166V	
	USH2A-05	USH2A c.6084T>A;p.Tyr2028Ter	USH2A c.11864G>A p.Trp3955Ter	
	USH2A-06	USH2A c.2276G>T;p.Cys759Phe	USH2A c.11864G>A;p.Trp3955Ter	yes
	USH2A-07	USH2A c.13335_13347delinsCTTG;p.Glu4445_Ser4449delinsAspLeu	USH2A c.10974_10975dup;p.Thr3659IlefsTer16	yes
	USH2A-08	USH2A c.11864G>A;p.Trp3955Ter	USH2A c.8522G>A;p.Trp2841Ter	
	USH2A-09	USH2A c.3381del;p.Thr1128ProfsTer10	USH2A Deletion Exons 22-24	
	USH2A-10	USH2A c.7950dup;p.Asn2651GlnfsTer10	USH2A c.15063_15081delinsGC;p.Thr5022GlnfsTer150	yes
	USH2A-11	USH2A c.11105G>Ap.Trp3702Ter	USH2A c.920_923dup;p.His308GlnfsTer16	yes

Mutation type	Subject ID	Variant 1	Variant 2	Segregation analysis available
XL-RPGR	XLRP-01	RPGR c.2416G>T;p.Glu806Ter		
	XLRP-03	RPGR c.2426_2427del;p.Glu809GlyfsTer25		
	XLRP-04	RPGR c.2590G>T;p.Glu864Ter		
	XLRP-05	RPGR c.2405_2406del;p.Glu802GfsTer32		
	XLRP-06	RPGR c.296C>A;p.Thr99Asn		
	XLRP-07	RPGR c.2442_2445del;p.Gly817LysfsTer2		
	XLRP-08	RPGR c.2543del;p.Glu848GlyfsTer241		
	XLRP-09	RPGR c.2527dup;p.Glu843GlyfsTer236		
	XLRP-10	RPGR c.2405_2406del;p.Glu802GlyfsTer32		
	XLRP-11	RPGR c.1217dup;p.Ser407IlefsTer46		
	XLRP-12	RPGR c.2568dup;p.Lys857GlufsTer222		
	XLRP-13	RPGR c.2323_2324del;p.Arg775GlufsTer59		yes
	XLRP-14	RPGR c.2384del;p.Glu795GlyfsTer20		
	XLRP-15	RPGR c.2236_2237del;p.Glu746ArgfsTer23		

Supplementary Table S1 Detailed information of genetic variants
